# Editorial: Moral psychology of AI

**DOI:** 10.3389/fpsyg.2024.1382743

**Published:** 2024-03-11

**Authors:** Feng Yu, Chris Krägeloh, Jaishankar Bharatharaj, Xiaojun Ding

**Affiliations:** ^1^Department of Psychology, Wuhan University, Wuhan, China; ^2^PAIR Lab, Department of Psychology and Neuroscience, Auckland University of Technology, Auckland, New Zealand; ^3^PAIR Lab, Bharath Institute of Higher Education and Research, Chennai, India; ^4^Department of Philosophy, School of Humanities and Social Sciences, Xi'an Jiaotong University, Xi'an, China

**Keywords:** moral psychology of AI, moral judgment, robopsychology, mind uploading, uncanny valley, autonomous vehicle, human-robot interaction, AI algorithm

The recent advancements in Artificial Intelligence (AI) have significantly expanded technological boundaries, prompting an urgent re-assessment of ethical guidelines and AI's role in science in order to address the more complex interactions with these advanced systems (Krägeloh et al., 2022; Ladak et al., 2023). Robopsychology and other emerging fields that provide a nexus of ethics, cognitive science, and AI, critically examine AI's capabilities to perceive, learn, and make decisions that carry moral weight. As AI matures, dissecting its ethical implications is imperative, paralleling the importance of its technical innovations and signaling a pivotal juncture in the discourse on AI (Xu et al., 2022; Zhang et al., 2023; Bonnefon et al., 2024).

The Research Topic “*Moral psychology of AI*” probes the nuanced interplay between ethically-aligned AI and its assimilation into society, evaluating how AI converges with human moral constructs. The studies included in this issue address a range of topics, from the ethical frameworks in autonomous vehicle algorithms and moral perceptions in human-robot interactions, to the fairness of AI in education, the impact of robot aesthetics on moral judgments, and the existential implications of speculative technologies like mind uploading. This editorial synthesizes five important papers that broaden our comprehension of ethical AI, dissecting the intricate relationship between AI and moral principles.

Sui established a benchmark for public preferences in moral algorithms for autonomous vehicles (AVs), probing the ethical frameworks guiding their critical decision-making. Utilizing a survey of 460 Chinese participants about the so-called trolley problem, the study contrasts Utilitarianism, Rawlsianism, Egoism, and a Hybrid model in AV ethics, uncovering a tension between algorithmic preference and purchase intent. While the study shows over half of the respondents' reluctance to purchase AVs equipped with an “egoism” algorithm, it revealed preference for a Hybrid model underscoring the complexity of aligning moral preferences with AI design. This contributes to the moral psychology narrative by identifying ethical priorities for AI applications.

Chen et al. progressed from Sui's foundational work to examine the dynamics of morality and reputation in human-robot interaction, crucial for collaborative potential. Their investigation demonstrates that humans apply moral and reputational considerations to robots, although distinct from human interactions. Through a series of three experiments, the study elucidates how reputation influences the interplay between moral considerations and sharing behavior, varying with the agent's nature—robotic or human. This research is instrumental for crafting conductive human-robot collaboration, appreciating the influence of moral and reputational perceptions in teamwork dynamics.

Progressing to practical AI applications, Chai et al. investigated AI evaluators in education. The study, with 466 participants, suggests students view AI as fairer than human teachers due to transparency. However, this perception aligns with human evaluators when AI's decisions are elucidated. This links AI ethics with transparency and the necessity for feedback, resonating with the moral preferences and trust issues in earlier studies by Sui and Chen et al.. It also touches upon ethical dilemmas like privacy and algorithmic dependence, emphasizing the delicate equilibrium of AI in educational ethics.

Laakasuo pivoted to the influence of robot aesthetics on human moral judgments. Findings indicate that robots with human-like appearances are treated more leniently for utilitarian actions, while “creepy” robots align better with deontological choices—determined through photorealistic depictions. This challenges prior research and highlights the role of visual design in moral psychology, advocating for consistent imagery in AI ethics studies.

Expanding the moral discourse, Laakasuo et al. explored the moral implications of speculative technologies like mind uploading. The investigation, featuring 1,007 participants, uncovers a correlation between existential beliefs and moral stances on mind uploading, with those valuing existential mattering and afterlife beliefs showing less moral support for the concept. This paper intersects technology and immortality with personal beliefs, suggesting mind uploading prompts a reevaluation of human existence and afterlife, intersecting religious beliefs, death anxiety, and the embrace of AI as a secular promise of immortality.

Collectively, these papers trace a thematic journey from AI's general moral preferences to specific applications (autonomous vehicles, education, human-robot interaction), the impact of physical robot design on moral judgments, and ultimately, the profound existential questions AI poses. This anthology of research intricately ventures into the nuanced interplay between AI and moral reasoning, positing a future where AI not only supplements but also sharpens human ethical judgments. Bridging theoretical discourse with empirical analysis, these publications lay a cornerstone for evolving toward an era where AI systems are intrinsically infused with ethical principles that resonate with societal values and human morality. The overarching aim is to harmonize AI's trajectory with our ethical compass, underscoring the criticality of fostering AI that is developed conscientiously and with ethical clarity.

All in all, this research initiative signals the commencement of a crucial conversation on reframing our ethical paradigms to keep pace with AI's evolution. This compilation will spark continued research and wider discourse on the ethical implications of AI, contemplating the ways in which AI reflects and reshapes our moral fabric, trust dynamics, and societal structures. It highlights the imperative for ethically conscious AI design, exploring themes of public trust, developer accountability, and the broader societal reverberations of AI incorporation. Future scholarly endeavors should seek to inspire ongoing inquiry of AI-related topics in a broader range of relevant fields such as psychology and other disciplines (Krägeloh et al., 2023), aspiring to craft AI that is both transparent and congruent with human values and ethics (Gabriel, 2020; Xu and Yu, 2020).

## Author contributions

FY: Writing—original draft. CK: Writing—review & editing. JB: Writing—review & editing. XD: Writing—original draft.
